# Alternating pressure air mattresses in the intensive care unit as a cost-effective strategy for preventing stage III–IV pressure injuries: a retrospective cohort study

**DOI:** 10.1016/j.clinsp.2026.100877

**Published:** 2026-03-06

**Authors:** Serdar Efe, Coşkun Ateş, Mehmet Serdar Cengizhan, Pervin Hancı, Volkan İnal

**Affiliations:** a Department of Intensive Care Medicine, Faculty of Medicine, Uludağ University, Bursa, Türkiye; b Department of Endocrinology Clinic, Çorlu City Hospital, Tekirdağ, Türkiye; c Department of Intensive Care Medicine, Tarsus State Hospital, Mersin, Türkiye; d Division of Intensive Care Medicine, Department of Pulmonology, Trakya University Faculty of Medicine, Edirne, Türkiye; e Division of Intensive Care Medicine, Department of Internal Medicine, Trakya University Faculty of Medicine, Edirne, Türkiye

**Keywords:** Pressure ulcer prevention, Alternating pressure air mattress, Cost-Effectiveness, ICU, NNT

## Abstract

**Background:**

Pressure Injuries (PIs) are frequent, preventable complications in Intensive Care Units (ICUs), leading to morbidity, mortality, and increased healthcare costs. Alternating Pressure Air Mattresses (APAMs) have been proposed to reduce PI incidence, but real-world data remain scarce.

**Objectives:**

To assess the effectiveness and cost-efficiency of APAMs in preventing PIs among critically ill patients.

**Methods:**

This retrospective cohort study was conducted in a 10-bed tertiary ICU, comparing patients before and after APAM implementation. Multivariate logistic regression identified independent PI risk factors, and a cost-effectiveness model was developed using Number Needed to Treat (NNT) and treatment costs.

**Results:**

APAM use reduced overall PI risk by 33% (RR = 0.67; 95% CI 0.45–0.98) and Stage III–IV ulcer risk by 84% (RR = 0.16; 95% CI 0.05–0.53). The NNT was 23, corresponding to net savings of USD 121,750 over five years for a 10-bed ICU, with cost recovery achieved within 4.3-months. Length of Stay (LOS) and prolonged mechanical ventilation independently increased PI risk by 19% and 13% per day, respectively. APAMs’ benefit was attenuated in patients with extended LOS and ventilation. Sepsis and respiratory failure increased PI risk in univariate analysis but lost significance after adjustment, indicating mediation by LOS and ventilation. APAMs eliminated the need for negative pressure wound therapy in this cohort.

**Conclusions:**

APAMs significantly reduced severe PI incidence and treatment requirements in critically ill patients, demonstrating cost-effectiveness. Their protective effect was limited in patients with prolonged ICU stay or ventilation. Multicenter randomized trials are warranted, especially in diabetic and septic populations.

## Highlight

• Alternating Pressure Air Mattresses (APAMs) cut Stage III–IV PI risk by 84 %.
• Cost recovery achieved in 4.3-months, saving USD 121,750 over 5-years.
• APAMs eliminated the need for negative pressure wound therapy in ICU.

## Introduction

Pressure Ulcers (PUs) are largely preventable yet common complications in Intensive Care Units (ICUs), contributing to increased morbidity, mortality, prolonged hospitalization, and significantly higher healthcare costs. The incidence of PU development is widely recognized as a key quality indicator in ICU settings. Despite the clinical relevance, evidence regarding the preventive efficacy of Alternating Pressure Air Mattresses (APAMs) in high-risk critically ill patients remains inconclusive. Some reports suggest a potential benefit, while others indicate no significant advantage. Current evidence does not strongly support the superiority of APAMs over other support surfaces in preventing pressure injuries.^1^

According to the updated 2025 guidelines of the National Pressure Injury Advisory Panel (NPIAP), the use of APAMs in patients at risk of pressure injury is recommended with a very low level of evidence (Grade 3A).^2^ Only a limited number of comparative studies have investigated the effectiveness of APAMs specifically in ICU populations. One earlier study from 2006, which included 221 mechanically ventilated patients, reported a reduction in PU incidence (18.67 vs. 12.41 cases per 1000 ICU days) with the use of APAMs.^3^ However, a more recent meta-analysis of four randomized controlled trials involving a total of 3308 ICU patients found no statistically significant difference in pressure injury prevention between Low Air Loss (LAL) systems and APAMs (8.9 % vs. 10.9 %, RR = 0.64).^4^

While it is generally recommended to select support surfaces based on individual patient characteristics and clinical needs, this tailored approach is often impractical in the dynamic and resource-constrained ICU environment. Additionally, the high cost of dynamic air mattresses, such as APAMs, may pose a significant barrier to widespread implementation, particularly in resource-limited settings. Therefore, selecting cost-effective support surfaces is essential for the optimal allocation of healthcare resources. Notably, data regarding the cost-effectiveness of routine APAM use in ICUs are scarce.

In this study, the authors aimed to evaluate the clinical effectiveness of APAMs in preventing pressure injuries among critically ill ICU patients and to conduct a cost-effectiveness analysis of their routine use.

## Methods

This retrospective cohort study was conducted in a 10-bed adult ICU of a tertiary care hospital, where the nurse-to-patient ratio was 1:3. The study was approved by the Ethics Committee of Trakya University Faculty of Medicine Hospital (Decision no 182/2025, dated 2 May 2025). Adult patients (aged ≥ 18-years) admitted to the ICU between 2 May 2016 and 2 May 2018 were evaluated. Patients admitted prior to 2 May 2017 comprised the control group (*n* = 375), while those admitted after the implementation of Alternating Pressure Air Mattresses (APAMs) on 2 May 2017 constituted the intervention group (*n* = 471).

Patients with spinal instability, defined as American Spinal Injury Association (ASIA) Impairment Scale grade A or B or radiographic evidence of vertebral fracture/dislocation, those weighing over 180 kg (due to device limitations), and pregnant women were excluded from the study.

All ICU beds in the intervention group were equipped with the same APAM model (Prima Series® 8000T, Taiwan), which provides dynamic pressure redistribution and simultaneous lateral rotation. The system utilizes a 20-cell horizontal air tube structure that cyclically inflates and deflates pressure zones in 8-minute intervals. The mattress also features a “static mode” designed for use in postoperative patients. According to the manufacturer, the average lifespan of the mattress pump system is five years.

The unit cost of the APAM was based on the 2025 manufacturer's price list. The selected system functions as an active support surface that continuously redistributes interface pressure by frequently alternating pressure points, thereby reducing the risk of pressure injury development in immobilized patients.

Prior to the intervention, all intensive care beds were equipped with Viscoelastic Foam Mattresses (VFM), and static air mattresses were used for cases with stage II and above PIs. All patients received standard PI prevention interventions (positioning, barrier cream use, twice-daily care, weekly full-body baths, and position changes as much as possible). The APAMs application was activated in continuous dynamic mode for 7 days/24 hours. The right/left positioning angle was kept at maximum (30 degrees). Static mode was preferred for postoperative patients, and dynamic mode was activated for patients who could not be transferred from intensive care to the ward when the postoperative hospitalization period exceeded three days.

The demographic data of all patients included in the study, comorbidities that could increase PI risk (diabetes, cancer, sepsis, respiratory failure), Ventilator Length of Stay (VLOS), and Length of Stay (LOS) were recorded. Electronic patient records (Hospital Information Management System) and nurse's ‘Pressure ulcer monitoring forms’ were used as data sources. PI stages present at admission and stage changes during follow-up (according to NPUAP/EPUAP classification) were recorded for patients in both groups. Additionally, the localisation and stages of newly developed PIs were also recorded.

### Statistical analysis

Descriptive statistics were presented as frequencies and percentages for categorical variables, and as mean ± standard deviation or median with Interquartile Range (IQR) for continuous variables, depending on the distribution. The normality of continuous variables was assessed, and comparisons between groups were performed using the Student's *t*-test for normally distributed variables and the Mann-Whitney U test for non-normally distributed variables. Categorical variables were compared using the Chi-Square test or Fisher’s exact test, as appropriate.

To identify factors independently associated with the development of pressure ulcers, univariate and multivariate logistic regression analyses were conducted. The multivariate model was adjusted for potential confounding variables, including age, presence of respiratory failure, diabetes mellitus, sepsis, and ICU length of stay. Statistical significance was defined as *p* < 0.05, and all results were reported with 95 % confidence intervals (95 % CIs).

Due to the retrospective nature of the data and the need for subgroup analyses, a post hoc power analysis was conducted. Using G*Power version 3.1, the calculated post hoc power was 68 % for an alpha level of 0.05, given the observed effect size (RR = 0.63) and the available sample size (*n* = 846).

Receiver Operating Characteristic (ROC) curve analysis was performed to determine the optimal cutoff values for relevant variables, using the Youden index (J = sensitivity + specificity - 1) to identify thresholds that maximize diagnostic performance. Statistical analyses were conducted using IBM SPSS Statistics for Windows, version 25.0 (IBM Corp., Armonk, NY, USA).

This study was reported in accordance with the STROBE (Strengthening the Reporting of Observational Studies in Epidemiology) statement for observational cohort studies. A completed STROBE checklist is provided as Supplementary Material.

## Results

After excluding eight patients (aged < 18 years, *n* = 2; spinal instability, *n* = 3; pregnancy, *n* = 2; weight > 180 kg, *n* = 1), a total of 846 patients were included in the final analysis (Control group: *n* = 375; APAM group: *n* = 471). No significant differences were observed in baseline demographic or clinical parameters, although pressure injury outcomes differed significantly between groups (Table 1).

**Table 1:** Demographic/Clinical characteristics and pressure ınjury outcomes: control and alternating pressure dynamic air mattresses.

Characteristic	Control Group (*n* = 375)	APAMs Group (*n* = 471)	p
**Demographic**			
Male, n ( %)	55.3 %	56 %	0.888^χ2^
Age, years^a^	64.1 ± 1.5 / [58‒72]	61.1 ± 1.1 / [54‒68]	0.067^m^
**Comorbidities**			
Diabetes ( %)	16.3 %	18.7 %	0.338χ²
Sepsis ( %)	29.7 %	37.2 %	0.122χ²
**Clinical Scores**			
APACHE-II score^b^	18.3 ± 9.2 / 17 [13]	20.02 ± 9.4 / 9 [14]	0.068^m^
SAPS- III score^b^	66.0 ± 28.5 / 65 [46]	68.0 ± 25.2 / 68 [40]	0.180^m^
**Care Duration Metrics**			
LOS, days^b^	10.1 ± 21.9 / 3 [8]	8.62 ± 19.5 / 2 [6]	0.260^m^
VLOS, days^b^	12.3 ± 25.6 / 5 [10]	9.87 ± 22.2 / 4 [8]	0.320^m^
**Outcomes**			
All Stages PIs (I‒IV), %	13.1 %	8.8 %	***0.037***χ²
Stage I‒II PIs	9.1 %	8.1 %	0.580χ²
Stage III‒IV PIs	4.0 %	0.64 %	*0.002*^f^
Pressure sore development day^c^	5 [3‒10]	6 [4‒12]	0.288^m^

APAMs, Alternating Pressure dynamic Air Mattresses; Pıs, Pressure Injuries; APACHE II, Acute Physiology and Chronic Health Evaluation II; SAPS-III, Simplified Acute Physiology Score III; LOS, Length of Stay (days); VLOS, Ventilator Length of Stay(days); SD, Standard Deviation; IQR, Interquartile Range.

χ² Ki-kare Test

m Mann-Whitney *U*.

f Fisher’s Exact Test.

a Mean ± SD/median [IQR = Q1-Q3].

b Mean ± SD/median [IQR].

c Median [IQR = Q1‒Q3]. Missing data: 〈 3 % for all variables; Bold/Italic: *p* < 0.05. Inclusion criteria: ICU patients age ≥ 18. Exclusion criteria: Age < 18, spinal instability, pregnant, weight > 180 kg. APAMs protocol: 8-minute cycles at 30‒45 mmHg pressure.

APAMs was associated with a median reduction of one day in both VLOS (5 vs. 4 days) and overall LOS (3 vs. 2 days). Conversely, the median day of new Pressure Injury (PI) development was one day later in the APAM group (5 vs. 6 days). However, these differences did not reach statistical significance.

**Fig. 1:**
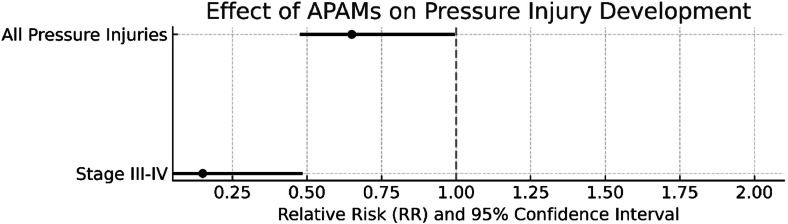
Effect of Alternating Pressure Air Mattresses (APAMs) on Pressure Sores: Relative Risk and 95 % Confidence Intervals Effect of APAMs on Pressure Injury Development; The horizontal line (RR = 1) represents the reference value. Confidence intervals below 1 support statistical significance.

The incidence of pressure injuries decreased from 13.1 % to 8.8 % following the implementation of APAMs, corresponding to an absolute risk reduction (ARR) of 4.3 %. Relative risk (RR) analysis indicated a 33 % reduction in the risk of developing a pressure injury among patients receiving APAMs (RR = 0.67; 95 % CI: 0.45–0.98). Furthermore, the risk of developing Stage III–IV pressure injuries was reduced by 84 % in the intervention group (RR = 0.16; 95 % CI: 0.05–0.53) (Fig. 1).

It was estimated that one pressure injury could be prevented for every 23 patients treated with alternating pressure air mattresses (Number Needed to Treat, NNT = 23). A 3.4 % absolute reduction in Stage III–IV PI incidence, the primary cost driver, was observed in the intervention group.

In the control group, 10 patients required extensive sacral debridement due to Stage IV pressure injuries, and 6 of them subsequently received negative pressure wound therapy (NPWT). In contrast, during the period when APAMs were used, only 3 patients required surgical debridement, and none required NPWT.

The most frequent PI sites in the APAM group were the presacral area (*n* = 25), back (*n* = 12), and gluteal region (*n* = 5). Less common sites included the heel (*n* = 3), scapula (*n* = 2), leg (*n* = 1), and shoulder (*n* = 1). Notably, 20 % of patients with pressure injuries had multiple lesions (≥ 2 ulcers).

At ICU admission, 5.1 % of patients in the APAM group already had existing pressure injuries. Among these, 3 patients progressed to Stage III/IV, 6 remained stable, 10 showed downgrading of ulcer stage, and in 5 patients, the pressure injuries fully healed during their ICU stay.

Subgroup analyses revealed a general trend toward reduced PI incidence in high-risk patient populations, with the exception of those with sepsis (Table 2). However, these reductions did not reach statistical significance. The authors believe this may be attributable to the limited statistical power of the study, which was calculated as 68 % in the post hoc analysis. Larger sample sizes may be required to detect statistically meaningful differences in future studies.

**Table 2:** Comparison of pressure injury incidence between Control and Alternating pressure dynamic air mattresses groups in high-risk subgroups.

Risk Factor^a^	Control (*n* = 375)	APAMs (*n* = 471)	RR (95 % CI)	Absolute risk reduction	p
APACHE II	13.1 %	12.3 %	1.07 (0.85‒1.35)	0.8 %	*0.056*χ²
≥ 20	12.8 %	10.3 %	0.78 (0.31‒1.92)	2.5 %	0.640^f^
Age > 65	15 %	8.5 %	0.38 (0.07‒2.15)	6.5 %	0.353^f^
Diabetic	13.8 %	17.3 %	1.30 (0.44‒3.83)	−3.5 %	0.794^f^
Septic	47 %	42.0 %	0.83 (0.35‒1.97)	5.0 %	0.826^f^
LOS > 7days	13.1 %	12.3 %	1.07 (0.85‒1.35)	0.8 %	*0.056*χ²
VLOS > 4 days	33.3 %	27.8 %	0.84 (0.52‒1.92)	5.5 %	0.640^f^

a High-risk subgroups defined a priori based on previous literature. APAMs, Alternating Pressure dynamic Air Mattresses; APACHE II: Acute Physiology and Chronic Health Evaluation II; LOS, Length of Stay; VLOS, Ventilator Length of Stay; RR, Relative Risk; CI, Confidence Intervals.

χ² Chi-Square test.

f Fisher's exact test. Missing data: < 3 % for all subgroups.

The incidence of pressure injuries was notably higher in patients with prolonged hospital stays (> 7 days) and those who underwent extended mechanical ventilation (> 4 days), compared to other risk subgroups.

**In the logistic regression analysis performed to identify independent risk factors associated with pressure injury development, both LOS and VLOS emerged as significant predictors (** Table 3 **).**

**Table 3:** Predictors of new pressure ınjury development: univariate and multivariate logistic regression.

Predictor	Univariate^LR^ OR (95 % CI)	p	Multivariate^LR^ aOR (95 % CI)	p
Air mattress	0.65 (0.33‒1.28)	0.207	a	a
Age	1.02 (0.99‒1.04)	0.077	1.03 (0.99‒1.07)	0.077
APACHE-II	0.91 (0.61‒1.01)	0.609	a	a
Saps-III	0.98 (0.98‒1.01)	0.078	1.02 (0.99‒1.04)	0.093
Diabetes	0.85 (0.34‒2.11)	0.720	a	a
Malignancy	0.93 (0.47‒1.81)	0.823	a	a
Respiratory failure	*1.98 (1.01‒3.85)*	*0.045*	1.70 (0.24‒2.06)	0.520
Sepsis	*2.69 (1.30‒5.28)*	*0.004*	1.73 (0.56‒4.99)	0.311
LOS	*1.17 (1.12‒1.22)*	*<0.001*	*1.19 (1.13‒1.25)*	*<0.001*^b^
VLOS	*1.13 (1.08‒1.17)*	*<0.001*	*1.13 (1.08‒1.17)*	*<0.001*^b^

LR aOR, Logistic Regression; OR, Odds Ratio; aOR, adjusted Odds Ratio; CI, Confidence Interval; APACHE II, Acute Physiology and Chronic Health Evaluation II; SAPS-III, Simplified Acute Physiology Score III; LOS, Length of Stay; VLOS, Ventilator Length of Stay. *Bold values indicate*: Statistical significance at *p* < 0.05.

a Values above *p* > 0.2 were not included in the Multivariate analysis.

b Both LOS and VLOS showed statistically significant associations with pressure ulcer risk in univariate and separate multivariate models (*p* < 0.01). Due to multicollinearity between variables, these were analyzed in distinct models. VLOS and LOS variables VIF = 2.81 (< 5) and Tolerance = 0.356 (> 0.1) [Moderate correlation]. Missing data handled with multiple imputation (*n* = 12, 1.4 %).

Both LOS and VLOS were identified as independent predictors of pressure injury development in separate multivariate logistic regression models (both *p* < 0.001). However, in a combined model including both variables, the independent predictive value of VLOS was no longer statistically significant.

**Fig. 2:**
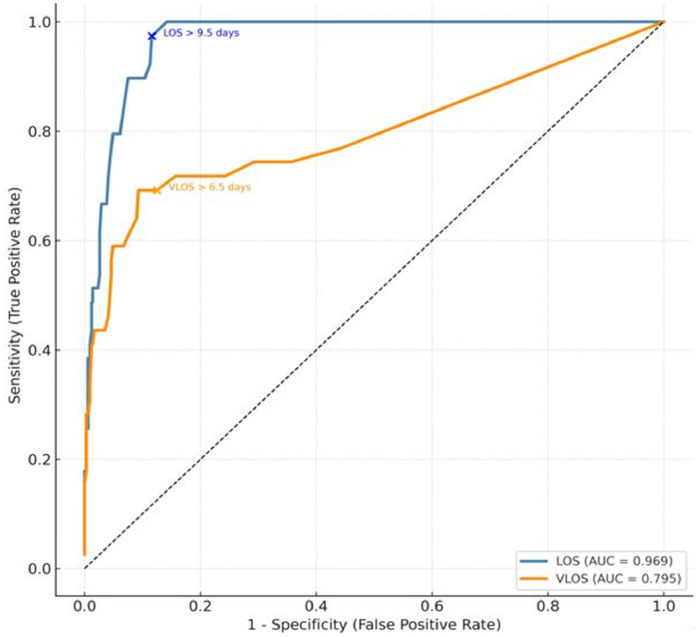
Performance of Intensive Care Length of Stay (LOS) and Mechanical Ventilation Duration (VLOS) in predicting pressure sore development: ROC curve analysis.

Receiver Operating Characteristic (ROC) curve analysis demonstrated that LOS had a significantly superior discriminative performance (Area Under the Curve [AUC = 0.969], Youden’s J = 0.810) compared to VLOS (AUC = 0.795, Youden’s J = 0.567) (Fig. 2). Optimal cutoff values were determined by maximizing the Youden index; for LOS, the threshold was 9.5 days (sensitivity 92.3 %, specificity 88.7 %), and for VLOS, 6.5 days (sensitivity 69.2 %, specificity 87.5 %). This ROC-based thresholding allows ICU clinicians to identify critical LOS (> 9.5 days) and VLOS (> 6.5-days) durations beyond which pressure injury risk rises exponentially.

Furthermore, risk increment analyses from separate models revealed that each additional day of LOS increased the risk of pressure injury by 19 % (*p* < 0.001), while each additional day of VLOS was associated with a 13 % increased risk (*p* < 0.001). Daily risk increments were estimated using separate regression models for LOS and VLOS, rather than day-by-day models, as suggested in time-to-event analyses.

The risk of developing pressure injuries was 2.7 times higher in patients with sepsis and 2 times higher in those with respiratory failure. Although respiratory failure and sepsis were identified as significant risk factors in univariate analysis, their significance was lost in the multivariate model. Age and SAPS III scores demonstrated borderline significance in the multivariate analysis.

## Discussion

This tertiary ICU study provides real-world evidence demonstrating that the use of Alternating Pressure Air Mattresses (APAMs) was associated with a reduction in the incidence of PIs in critically ill patients, although this association did not reach statistical significance in the multivariate analysis. APAM use was associated with a 33 % reduction in overall PI risk (RR = 0.67; 95 % CI: 0.45–0.98) and an 84 % decrease in Stage III–IV ulcers (RR = 0.16; 95 % CI: 0.05–0.53), indicating their potential efficacy in preventing deep tissue damage. The intervention group showed a marked decrease in the need for surgical debridement and negative pressure wound therapy. The Number Needed to Treat (NNT) of 23 to prevent one PI case translates into an estimated annual cost saving of approximately USD 24,000, highlighting the clinical and economic significance of APAM implementation. However, no protective effect was observed in Stage I–II ulcers (*p* = 0.580), suggesting that superficial injuries may involve additional risk factors beyond pressure redistribution. Importantly, most previous trials focused on all-stage pressure injuries, while the present analysis isolated severe (Stage III–IV) cases, providing targeted insight into the subset with the greatest clinical and economic impact.

The PI incidence in the control group (13.1 %) aligns with Australian ICU data (10.4 %–13.5 %).^5^ In the United States, hospital-acquired PIs cost approximately USD 26.8 billion annually, with about 60 % attributable to advanced-stage ulcers.^6^ The present study demonstrates that APAM use reduces Stage III–IV PI incidence from 4 % to 0.64 % (RR = 0.16; 95 % CI: 0.05–0.53), providing real-world evidence for potential cost burden reduction.

A hypothesis-driven cost-effectiveness model was developed based on literature reporting an average treatment cost of approximately USD 1500 per Stage III–IV ICU-acquired PI.^7^^,^^8^ Using the present data (NNT = 23; absolute Stage III–IV incidence reduction from 4.0 % to 0.64 %), a five years mattress lifespan, an annual patient load of 400, and a 10-bed ICU assumption, the authors estimated prevention of 87 pressure injuries over five-years. This yields a gross saving of USD 130,500 (87 cases × USD 1500). After deducting the APAM investment cost (USD 8750), net savings are projected at USD 121,750, equating to an average annual saving of approximately USD 24,350. Although promising, these projections may vary according to local cost structures and patient demographics.

Multivariate analyses identified LOS and VLOS as the strongest independent predictors of PI development. Each additional hospital day increased PI risk by 19 % (OR = 1.19; 95 % CI: 1.12–1.22), and each additional day of mechanical ventilation raised risk by 13 % (OR = 1.13; 95 % CI: 1.08–1.17). The substantial clinical burden may partly explain why APAMs’ protective effect did not reach statistical significance in the regression model (OR = 0.65; *p* = 0.207). Prolonged immobilization impairs microperfusion, triggering tissue injury via pathophysiological pathways not entirely mitigated by mattress technology alone. These findings align with a study of 256 patients in Italian ICUs, where age, LOS, mechanical ventilation, and SAPS-II scores were also identified as PI risk factors.^9^ This study provides real-world confirmation of these well-established risk factors within a high-risk tertiary ICU population.

While Norton and Braden scales remain traditional tools for early identification of high-risk patients, machine learning models show promise. Zheng et al. employed a SHAP-XGBoost algorithm on 29,448 ventilated patients in the MIMIC-IV database, achieving high accuracy in PI risk prediction (AUC = 0.86).^10^ These technologies are critical given that VLOS is not only a predictor of pressure injury but also a quality indicator correlating with mortality.^11^ Reducing ventilation duration is expected to decrease PI incidence and improve ICU outcomes.

Diabetes was not an independent risk factor for PI development in the critically ill population. Previous studies have reported conflicting results regarding the relationship between diabetes and PI risk. Frankel et al. (2007) and Gou et al. (2023) found diabetes increased PI risk 2.7–3.2 fold in surgical ICU and device-related ICU PI populations, respectively.^12^^,^^13^ However, Efteli and Güneş (2020), in a neurology ICU cohort, reported no significant association (*r* = 0.18).^14^ Although diabetes-related risk remains controversial, PI healing may be prolonged and complicated by infection in diabetic patients. Therefore, predictive and preventive efforts for PI in this subgroup remain clinically important, and machine learning models targeting diabetic critically ill diabetic patients are under development.^15^ The subgroup analysis showed a 6.5 % absolute risk reduction in PI incidence in diabetic patients receiving APAMs, though not statistically significant. Prospective randomized controlled trials focused on this subgroup are warranted.

In univariate analysis, sepsis (OR = 2.69; 95 % CI: 1.30–5.28; *p* = 0.004) and respiratory failure (OR = 1.98; 95 % CI: 1.01–3.85; *p* = 0.045) were significant risk factors for PI development, consistent with systematic data showing sepsis and septic shock increase PI risk two- to threefold.^16^^,^^17^ Notably, PIs can also serve as a primary source of bloodstream infections; Pınar et al. demonstrated increased mortality risk associated with PI-related infections.^18^ The apparent inefficacy of APAMs in septic patients (Table 2) may be explained by sepsis-induced microperfusion impairment and inflammatory cascades overriding the protective effects of pressure redistribution. Multimodal strategies combining APAMs with early goal-directed antibiotics, ω−3 supplemented nutrition, and passive mobilization within 48 h should be investigated in future studies.

Similarly, respiratory failure likely increases PI risk via hypoxia-induced tissue hypoperfusion and immobilization. However, both factors lost significance in multivariate analysis (sepsis: OR = 1.73, *p* = 0.311; respiratory failure: OR = 1.70, *p* = 0.520), suggesting their effects are largely mediated by LOS and VLOS. This supports the hypothesis that prolonged hospitalization and ventilation independently trigger tissue injury pathways beyond the primary clinical conditions.

In conclusion, while dynamic air mattresses represent an important supportive intervention, their effectiveness may be limited in the presence of risk factors such as prolonged LOS and mechanical ventilation. Hence, preventive strategies in high-risk patients should integrate multidisciplinary approaches, including early mobilization, prompt ventilator weaning, and sepsis control.

APAM intervention did not significantly reduce PI incidence in high-risk subgroups (APACHE II ≥ 20, age > 65, diabetes, LOS > 7 days, VLOS > 4 days) (Table 3), although a protective trend was observed in the APACHE II ≥ 20 group (*p* = 0.056). Relative risks below 1 in other subgroups (age > 65: RR = 0.78; diabetes: RR = 0.38; LOS > 7-days: RR = 0.83) suggest potential benefits, but retrospective design limitations and a post hoc power of 68 % explain the lack of statistical significance. Achieving 80 % power would require 554 patients per group. The 6.5 % absolute risk reduction in diabetics (NNT = 15) may be clinically relevant despite statistical insignificance.

### Strengths

Intervention standardization was ensured by using the same APAM model on all beds. The single-center historical control design preserved high internal consistency in patient population, nursing protocols, and environmental factors. Twelve months of data collection mitigated seasonal variation effects, enhancing the validity. This methodology provides high-quality real-world effectiveness data. Clinically relevant outcomes, such as the 84 % reduction in Stage III–IV ulcers (RR = 0.16) and NNT = 23, offer actionable evidence to update ICU protocols.

### Limitations

The retrospective design carries risks of measurement bias due to reliance on electronic health records, though missing data was < 3 %. The historical control approach may introduce confounding from temporal changes in nursing protocols or comorbidity distributions, partially adjusted by time-trend analysis. Single-center design with a relatively small sample limits generalizability. Absence of biomechanical parameters (e.g., transcutaneous oximetry, pressure mapping), and lack of covariates such as BMI, nutritional status, and humidity control, limit the evaluation of factors like the obesity paradox reported by Chen et al.^19^ The 68 % power in subgroup analyses increases the type II error risk in diabetic (*n* = 141) and septic (*n* = 175) patients. The cost model based on US treatment costs (∼USD 1500 per case) and fixed NNT requires local validation for other regions. Nonetheless, these findings generate hypotheses for future randomized controlled trials.

## Conclusion

This tertiary ICU study suggests that APAM use may reduce Stage III–IV PI incidence by 84 %, decrease the need for surgical debridement, and potentially eliminate the requirement for negative pressure wound therapy. The cost model projects a net saving of USD 121,750 over five years in a 10-bed ICU, with an initial investment payback period of 0.36 years (∼4.3-months). These findings provide Level B evidence (CEBM 2009 criteria) supporting the integration of APAMs into ICU PI prevention protocols. To enhance generalizability, adequately powered (*n* > 712) multicenter randomized trials in diabetic and septic subgroups are warranted.

## Availability of data and materials

The data supporting the findings of this study are available from the corresponding author upon reasonable request.

## Ethics approval and consent to participate

This study was approved by the Institutional Ethics Committee of Uludağ University Faculty of Medicine (Approval no 182/2025, Date :02.05.2025). Informed consent was waived due to the retrospective design.

## Approval for publication

As this study was retrospective in nature, informed consent was not obtained from patients or their relatives. However, the study was conducted in accordance with the ethical principles outlined in the latest version of the Declaration of Helsinki.

## Authors’ contributions

Serdar Efe: Conceptualization; formal analysis; ınvestigation; writing-original draft.

Coşkun Ateş: Conceptualization; data curation; methodology; writing-original draft.

Mehmet Serdar Cengizhan: Conceptualization; data curation; methodology; writing-original draft.

Pervin Hancı: Investigation; formal analysis; writing-review & editing.

Volkan İnal: Conceptualization; formal analysis; methodology; writing-original draft.

All authors read and approved the final manuscript.

## Funding

This research received no specific grant from any funding agency in the public, commercial, or not-for-profit sectors.

## Declaration of competing interest

The authors declare no conflicts of interest.

## Appendix Supplementary materials

Image, application 1
