# Supplementary material for: Alternating pressure air mattresses in the intensive care unit as a cost-effective strategy for preventing stage III–IV pressure injuries: a retrospective cohort study
Source: Clinics (Sao Paulo). 2026 Mar 6;81:100877. doi: 10.1016/j.clinsp.2026.100877 (PMC12993162; doi:10.1016/j.clinsp.2026.100877)
Supplement: Supplementary file 1 [file mmc1.docx]

| **STROBE Checklist for Retrospective Cohort Studies** | | |
| --- | --- | --- |
| **Article:** Alternating Pressure Air Mattresses in the Intensive Care Unit: A Cost-Effective Strategy for Preventing Stage III–IV Pressure Injuries: A Retrospective Cohort Study | | |
| **Section & Item** | **Item Description** | **Reported in Manuscript (Page/Line)** |
| **Title & Abstract** | Indicate the study’s design with a commonly used term in the title or abstract | Title: "A Retrospective Cohort Study" (Page 1, Line 2); Abstract: "This retrospective cohort study" (Page 3, Line 61) |
|  | Provide an informative and balanced summary of what was done and what was found | Abstract includes background, methods, results, conclusions (Pages 3–4, Lines 54–76) |
| **Introduction** | Explain the scientific background and rationale for the investigation | Introduction (Pages 4–5, Lines 81–105) |
|  | State specific objectives and hypotheses | Page 5, Lines 101-108 |
| **Methods** | Describe the setting, locations, and relevant dates | Page 5, Lines 111-117 |
|  | Give the eligibility criteria, and sources and methods of selection | Methods: inclusion/exclusion criteria (Pages 5–6, Lines 114–122) |
|  | Clearly define all outcomes, exposures, predictors, etc. | PI staging, APAM use, risk factors PI staging, APAM exposure, (Pages 6–7, Lines 124–151) |
|  | For each variable, give sources of data and details of assessment | Data sources: HIS and nurse pressure ulcer forms (Pages 6–7, Lines 146–148) |
|  | Describe any efforts to address potential sources of bias | Standardized care protocols, same APAM model (Page 14, Lines 274–275) |
|  | Explain how study size was arrived at | Post-hoc power analysis (Page 7, Lines 166–169) |
|  | Explain how quantitative variables were handled | Continuous variables as mean±SD or median (IQR) (Pages 7–8, Lines 155–165) |
|  | Describe all statistical methods | Statistical analysis section (Pages 7–8, Lines 153–165) |
| **Results** | Report numbers of individuals at each stage of study | Participant flow and exclusions described (Page 8, Lines 179–181) |
|  | Give characteristics of study participants | Baseline characteristics and outcomes (Table 1, Page 9) |
|  | Report numbers of outcome events | PI incidence before/after APAM implementation (Pages 10–11, Lines 197–216) |
|  | Give unadjusted and adjusted estimates | Logistic regression analyses (Table 3, Page 12, Lines 232–245) |
|  | Report other analyses | Subgroup analyses and ROC analyses (Pages 11–13, Lines 217–264; Table 2) |
| **Discussion** | Summarize key results with reference to objectives | Discussion (Page 14, Lines 286–290) |
|  | Discuss limitations, considering sources of bias | Limitations discussed (Page 14, Lines 273–275) |
|  | Give a cautious overall interpretation | Interpretation and implications (Page 14, Lines 281–285) |
| **Other** | Discuss generalizability  Source of funding and role of funders  Ethical approval and consent | Discussion (Page 14, Lines 286–290)  None declared (Page 2, Lines 36–37)  Ethics approval (Page 2, Lines 38–43) |
